# Molecular phylogenetic analyses support the monophyly of Hexapoda and suggest the paraphyly of Entognatha

**DOI:** 10.1186/1471-2148-13-236

**Published:** 2013-10-31

**Authors:** Go Sasaki, Keisuke Ishiwata, Ryuichiro Machida, Takashi Miyata, Zhi-Hui Su

**Affiliations:** 1 JT Biohistory Research Hall, 1-1 Murasaki-cho, Takatsuki, Osaka 569–1125, Japan; 2 Department of Biological Sciences, Graduate School of Science, Osaka University, Osaka 560-0043, Japan; 3 Sugadaira Montane Research Center, University of Tsukuba, Sugadaira Kogen, Ueda, Nagano 386-2204, Japan; 4 Present address: School of Medicine, Kumamoto University, Kumamoto 860-8556, Japan; 5 Present address: Division of Functional Genomics, Advanced Science Research Center, Kanazawa University, Kanazawa 920-0934, Japan

**Keywords:** Pancrustacea, Hexapoda, Entognatha, Molecular phylogeny, RNA polymerase gene, DNA polymerase gene

## Abstract

**Background:**

Molecular phylogenetic analyses have revealed that Hexapoda and Crustacea form a common clade (the Pancrustacea), which is now widely accepted among zoologists; however, the origin of Hexapoda remains unresolved. The main problems are the unclear relationships among the basal hexapod lineages, Protura (proturans), Collembola (springtails), Diplura (diplurans), and Ectognatha (bristletails, silverfishes, and all winged insects). Mitogenomic analyses have challenged hexapod monophyly and suggested the reciprocal paraphyly of Hexapoda and Crustacea, whereas studies based on nuclear molecular data support the monophyletic origin of hexapods. Additionally, there are significant discrepancies with respect to these issues between the results of morphological and molecular studies. To investigate these problems, we performed phylogenetic analyses of Pancrustacea based on the protein sequences of three orthologous nuclear genes encoding the catalytic subunit of DNA polymerase delta and the largest and second largest subunits of RNA polymerase II from 64 species of arthropods, including representatives of all hexapod orders.

**Results:**

Phylogenetic analyses were conducted based on the inferred amino acid (aa) sequences (~3400 aa in total) of the three genes using the maximum likelihood (ML) method and Bayesian inference. Analyses were also performed with additional datasets generated by excluding long-branch taxa or by using different outgroups. These analyses all yielded essentially the same results. All hexapods were clustered into a common clade, with Branchiopoda as its sister lineage, whereas Crustacea was paraphyletic. Within Hexapoda, the lineages Ectognatha, Palaeoptera, Neoptera, Polyneoptera, and Holometabola were each confirmed to be monophyletic with robust support, but monophyly was not supported for Entognatha (Protura + Collembola + Diplura), Ellipura (Protura + Collembola), or Nonoculata (Protura + Diplura). Instead, our results showed that Protura is the sister lineage to all other hexapods and that Diplura or Diplura + Collembola is closely related to Ectognatha.

**Conclusion:**

This is the first study to include all hexapod orders in a phylogenetic analysis using multiple nuclear protein-coding genes to investigate the phylogeny of Hexapoda, with an emphasis on Entognatha. The results strongly support the monophyletic origin of hexapods but reject the monophyly of Entognatha, Ellipura, and Nonoculata. Our results provided the first molecular evidence in support of Protura as the sister group to other hexapods. These findings are expected to provide additional insights into the origin of hexapods and the processes involved in the adaptation of insects to life on land.

## Background

The phylum Arthropoda consists of four major groups: Chelicerata, Crustacea, Myriapoda, and Hexapoda. Recent molecular analyses have greatly changed our traditional understanding of arthropod phylogeny and evolution. These studies have rejected the traditional view that the closest relatives to hexapods are myriapods, and instead indicate that hexapods and crustaceans form a common clade, which is now called Pancrustacea [1-13]. Pancrustacea is also supported by the mitochondrial gene order [14] and by studies of ultrastructure and neurogenesis of the eye and brain [15,16]. However, the origin of Hexapoda is still an open question, and the phylogenetic relationships among the basal hexapod lineages remain unclear despite the considerable research efforts that have conducted in attempts to resolve them (see reviews: [17-19]).

**Figure 1:**
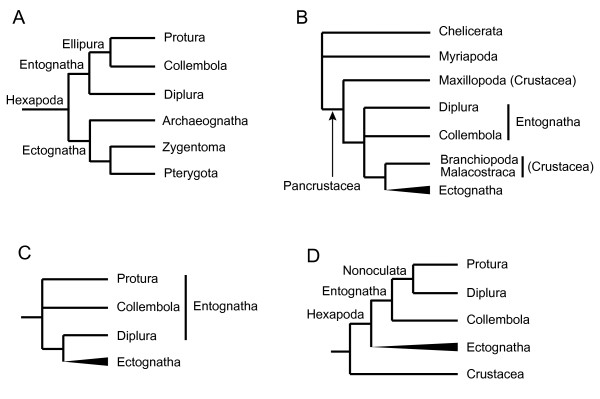
**The major hypotheses of the basal hexapod relationships proposed in recent studies. (A)** Traditional view based on morphology [20,49]. **(B)** Based on mitogenomic data [22,23]. **(C)** Based on fossil data [32], comparative embryological evidence [33,34], morphological data [28,35], and some molecular sequences (EF-1α, EF-2, and RAN polymerase II) [36]. **(D)** Based on nuclear molecular data [7,8,29,30,37].

The subphylum Hexapoda (Insecta *sensu lato*) is taxonomically classified into two major classes: Entognatha and Ectognatha (Insecta *sensu stricto*) [20]. Entognatha comprises three wingless orders, Protura (proturans), Collembola (springtails), and Diplura (diplurans); Ectognatha consists of two wingless orders (Archaeognatha [bristletails] and Zygentoma [silverfishes]), and all winged insects (Pterygota) (Figure 1A). Although hexapods are traditionally considered to be a monophyletic group [21], Nardi and colleagues [22,23] presented phylogenetic trees based on mitochondrial DNA sequences that indicated that collembolans and diplurans branched off much earlier than the separation between ectognathans and some crustaceans such as branchiopods and malacostracans, implying that hexapods are not monophyletic (Figure 1B). In support of this hypothesis, Cook et al. [24] analyzed mitogenomic data and suggested that hexapods and crustaceans may be mutually paraphyletic. The reciprocal paraphyly of Hexapoda and Crustacea means that hexapods have independently evolved at least twice from different crustacean-like ancestors, and that the six-legged body plan is the result of convergent evolution. However, the mitochondrial data have been indicated to potentially be misleading for the tree reconstruction of deep arthropod lineages [25-27]. Furthermore, phylogenetic analyses based on nuclear 18S and 28S ribosomal RNA data [6,7,13,28-30], and nuclear protein-coding genes [8,9,11,31] support the monophyletic origin of hexapods. These contradictory results have currently called much attention to the problem of the origin, phylogeny, and evolution of hexapods, and also to interpretations of the adaptation of insects to life on land.

The key to understanding the origin and adaptations of hexapods is the confirmation of (1) the monophyly or paraphyly of Hexapoda and (2) the phylogenetic relationships of entognathans. The reasoning behind the first point is described above. Regarding the second issue, studies on the basis of data from fossils [32], comparative embryological evidence [33,34], morphological data [28,35], and molecular sequences (EF-1α, EF-2, and RNA polymerase II) [36] suggest that Diplura is the closest relative of Ectognatha (Insecta), thereby making Entognatha paraphyletic (Figure 1C). However, analyses performed using rDNA sequences [7,29,30,37], combined molecular sequence and morphological data [38], and expressed sequence tag (EST) data [8] support the monophyly of Entognatha and suggest that a sister relationship exists between Protura and Diplura, *i.e.*, the arrangement of Collembola + (Protura + Diplura) (Figure 1D).

The major aim of this study was to investigate the phylogenetic relationships of Hexapoda, with an emphasis on basal hexapod lineages, using three nuclear genes encoding the catalytic subunit of DNA polymerase delta (DPD1) and the largest and second largest subunits of RNA polymerase II (RPB1 and RPB2, respectively), with extensive taxon sampling of all hexapod orders. The amino acid (aa) sequences of these proteins were used to perform phylogenetic analyses using the maximum likelihood (ML) method and Bayesian inference.

## Results

### Sequence and alignment dataset

In this study, the nuclear genes encoding DPD1, RPB1, and RPB2 were amplified and sequenced in 14 arthropods, which consisted of six species of Entognatha (one dipluran, three collembolans, and two proturans), six species of Crustacea (three branchiopods, two malacostracans, and one maxillopodan), one myriapod, and one chelicerate (Additional file 1). For DPD1, complete aa sequences (1092 to 1153 aa) were obtained for eight of these taxa, and nearly complete sequences (957 to 1006 aa) were determined for the remaining taxa (Additional file 2). For RPB1, the *C*-region (~400 aa) contains a repeated sequence that is not suitable for phylogenetic analysis; therefore, we did not determine the sequence of the 3’-terminal region of this gene. However, the 5’-terminal region was completely sequenced for all 14 taxa with the exception of one species (*Daphnia pulicaria*). Consequently, the sequence length of RPB1 used in the analyses ranged from 1516 to 1796 aa (Additional file 3). For RPB2, complete sequences (1169–1179 aa) were obtained for 12 of the taxa, and a small *N*-terminal region was missing from the sequence of the remaining two taxa (1146 and 1152 aa) (Additional file 4). The sequence data for ectognathan species determined in our previous study [39] and one chelicerate sequence (*Ixodes scapularis*) from the database were added to the entire dataset for the phylogenetic analyses. This generated a large sequence dataset covering a total of 64 arthropod species, including 55 hexapods representing all hexapod orders (Additional file 1). The aa sequences of each protein were aligned using MAFFT [40], and the unambiguously aligned sites (DPD1, 873 aa; RPB1, 1401 aa; RPB2, 1126 aa) selected from each alignment using Gblocks ver. 0.91b [41,42] were concatenated into a single alignment dataset with a total length of 3400 aa.

To understand the contributions of the individual markers to the phylogenetic analysis, the proportion of parsimony-informative and variable aa sites in the alignment of each protein was calculated with MEGA ver. 5.05 [43]. These results showed that the proportion of variable aa sites was 71.70% for DPD1, 44.75% for RPB1, and 36.51% for RPB2, and that the parsimony-informative proportions were 62.77%, 33.83% and 26.55%, respectively (Table 1). These results indicated that the three genes have different evolutionary rates: DPD1 evolves more rapidly than the other two, and RPB2 evolves the most slowly.

**Table 1:** Proportions of parsimony-informative and variable amino acid sites in the alignment dataset (64 OTU)

**Gene**	**Total**	**Parsim-info**	**Singleton**	**Variable**	**Conserved**	**Variable/total**	**Parsim-info/total**
DPD1	873	548	78	626	247	71.70%	62.77%
RPB1	1401	474	153	627	774	44.75%	33.83%
RPB2	1126	299	112	411	715	36.51%	26.55%
Total	3400	1321	343	1664	1736	48.37%	38.85%

### Phylogeny inferred from the complete dataset

Before performing phylogenetic analyses using the concatenated alignment, we performed separate analyses based on the alignments of the individual genes to detect whether there were significant discrepancies among the tree topologies. Although some discrepancies were found in the tree topologies, the nodes representing the incongruent relationships had low support except for two nodes with moderate support (Additional files 5 and 6). These two nodes, which were found in the DPD1 tree, formed obviously anomalous relationships, *Anacanthocoris striicornis* (Hemiptera) + Palaeoptera and *Uroleucon nigrotuberculatum* + *Cryptotympana facialis*, most likely due to long-branch attraction (LBA) artifacts. Overall, there were no significant conflicts in the tree topologies of the individual genes.

**Figure 2:**
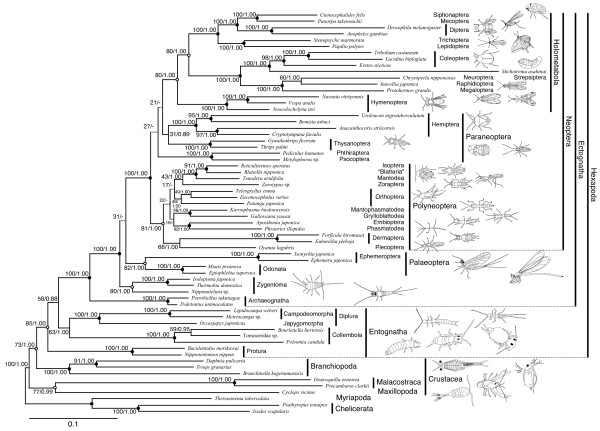
**The ML tree of pancrustaceans inferred from the amino acid sequences of DPD1, RPB1, and RPB2.** The branch lengths were calculated from the concatenated alignment of the three protein sequences. Bootstrap values and posterior probabilities are shown at nodes. Dot-marked nodes: bootstrap value > 90%, posterior probability = 1.00. Circle-marked nodes: bootstrap value 70%-90%, posterior probability = 1.00 (except node 59). Internal branches drawn as dotted lines: not supported by Bayesian analysis.

Based on the concatenated aa sequence alignment dataset of DPD1, RPB1, and RPB2, the phylogenetic analysis was performed on the RAxML and MrBayes programs. The ML tree topology is shown in Figure 2. Discrepancies in the tree topologies between the ML analysis and Bayesian inference were observed at five nodes whose internal branches were marked by dotted lines in the tree, but these nodes had very low supporting values in the analyses (Figure 2). Thus, there were no significant conflicts in the topologies based on the different analysis methods.

In the two trees resulting from the ML and Bayesian methods, the proturans, collembolans, diplurans, and all ectognathans (insects *sensu stricto*) clustered together to form the monophyletic group Hexapoda. Branchiopoda was revealed to be a sister lineage to Hexapoda, and Malacostraca was closely related to Copepoda (Class Maxillopoda), resulting in a paraphyletic Crustacea (Figure 2). On the other hand, the present analyses did not support the monophyletic origin of Entognatha. Instead, they showed that Protura is a sister lineage to other hexapods and that Collembola and Diplura form a common clade that is closely related to Ectognatha (Figure 2). In addition, the two suborders Japygomorpha and Campodeomorpha jointly formed a single clade with strong support (Figure 2), supporting the monophyly of Diplura. Within Ectognatha, the monophyly of Ectognatha, Palaeoptera, Neoptera, Polyneoptera, and Holometabola was well supported, but Paraneoptera did not form a single cluster. Archaeognatha was inferred to be a sister lineage to the other ectognathans, but the relationships among the three basal lineages of Neoptera (Polyneoptera, Paraneoptera, and Holometabola) were not resolved (Figure 2). Thus, which lineage is most closely related to holometabolous insects remains unclear. These results and the interordinal relationships of each higher taxonomic group were consistent with those from our previous study [39].

### Phylogeny inferred from selected datasets

To confirm the above findings, further phylogenetic analyses were performed using three selected datasets that were modified from the original by either excluding long-branch taxa, using crustaceans as an outgroup, or excluding collembolans.

The ML tree shown in Figure 2 contained several visually long branches, corresponding to *Drosophila melanogaster, Stichotrema asahinai, Uroleucon nigrotuberculatum, Anacanthocoris striicornis, Forficula hiromasai, Euborellia plebeja, Isonychia japonica*, and *Ephemera japonica*. To eliminate the possibility of LBA artifacts [44], we conducted phylogenetic analyses based on a dataset that excluded the sequence data of the long branches using the same analysis procedures. The resulting trees showed the same topology as those obtained from the original analysis, with the exception of the positions of two Paraneoptera orders (Phthiraptera and Psocoptera), which were weakly supported in the analyses (Additional files 7 and Figure 2). This result suggests that the long-branch taxa do not introduce LBA artifacts into the phylogenetic analyses.

**Figure 3:**
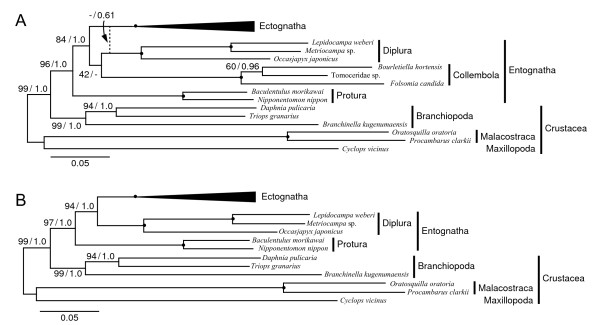
**Phylogenetic tree of Hexapoda using crustaceans as outgroups. (A)** With collembolans. **(B)** Without collembolans. Phylogenetic analyses were performed with RAxML and MrBayes. The bootstrap value and posterior probability are shown at each node. The topologies of Ectognatha are omitted in this tree. For the details of these analyses and the original trees, see Additional files 8, 9, 10 and 11.

The selection of an appropriate outgroup often improves the results of a phylogenetic analysis. In general, because outgroup taxa represent long branches, a given outgroup may cause the misplacement of long-branched ingroup taxa. Using an outgroup that is much more closely related to the ingroups may avoid such artifacts. Therefore, we conducted additional phylogenetic analyses using crustaceans as the sole outgroup because they are more closely related to hexapods than chelicerates and myriapods, and excluded sequences of chelicerates and myriapods from the full dataset. The analyses were performed with the same methods described above, and the results are shown in Figure 3A (for the original trees, see Additional files 8 and 9). Compared with the tree generated from the full dataset (Figure 2), the monophyletic origin of hexapods and the placement of Protura as a sister group to all other hexapods were supported much more strongly in all analyses (supporting values: 96/1.0 and 84/1.0, respectively) (Figure 3A). In contrast, the sister relationship between Collembola and Diplura was only supported by the RAxML analysis, whereas the analysis with MrBayes inferred that Diplura is a sister lineage to Ectognatha, although the support was weak (broken line in Figure 3A).

Finally, we repeated the phylogenetic analyses that excluded the sequence data of collembolans in an attempt to further confirm the sister relationship between Protura and Diplura, and determine whether the relatively long branch of the collembolans affects the relationships of the entognathans. The phylogenetic trees generated from these analyses are shown in Figure 3B, and the original trees are shown in Additional files 10 and 11. Despite the exclusion of the collembolans, these analyses still placed Protura as a sister group to the remaining hexapods and robustly supported (94/1.0) the close relationship between Diplura and Ectognatha. However, they did not support the clade Nonoculata, *i.e.*, no sister relationship between Protura and Diplura was suggested by these trees.

## Discussion

### Monophyletic origin of Hexapoda

Hexapods are traditionally considered to be monophyletic primarily based on their common body structure, consisting of a head, a thorax, an abdomen, and three pairs of thoracic legs. The monophyletic origin of Hexapoda was called into question by phylogenetic analyses based on mitochondrial genomic data that found wingless collembolans and diplurans to be more closely related to crustaceans than to other hexapods [22,23]. These studies suggested that hexapods and crustaceans are reciprocally paraphyletic. However, phylogenetic analyses based on nuclear molecular data, including rDNA [6,7,13,28-30] and protein-coding genes [8,9,11,31], have recovered Hexapoda as monophyletic. Our present results strongly support the monophyly of hexapods and the paraphyly of crustaceans (Figures 2 and 3). In contrast to preceding studies, our analyses included samples from all hexapod orders, and multiple species from each entognathan order (Protura, Collembola, and Diplura), thereby strengthening the reliability of our results on hexapod phylogeny.

Our present results, together with those of other recent phylogenetic analyses based on nuclear genomic data [8,11,29], indicate that Hexapoda is a monophyletic taxon. To confirm this conclusion, however, a reasonable interpretation of the contrasting results obtained from mitochondrial genomic data [22,23] is also needed. Several studies [25,26] indicated that it is difficult to resolve the relationships among the basal arthropod lineages using mitogenomic data alone because the relationships inferred by such data are highly influenced by the choice of the outgroup, data treatment method, and genes. However, following the study of Nardi et al. [22], analyses performed with a large mitochondrial genomic dataset and various analytical methods still suggested the reciprocal paraphyly of Crustacea and Hexapoda [23]. Therefore, the conflicting results of analyses conducted using mitochondrial and nuclear data may need to be explained by other factors, such as introgressive hybridization between crustaceans and hexapods in the early stages of hexapod diversification, or incomplete lineage sorting of ancestral polymorphisms in the mitochondrial genome. If any of these speculations are accurate, one may expect to find some nuclear genes that support the results of the phylogeny based on the mitogenomic data. Indeed, in the present analyses with the individual gene sequence data, some tree topologies did suggest the reciprocal paraphyly of Crustacea and Hexapoda, although the support was very weak (Additional files 5A and 6C). Given this information, considering the results inferred from the combined large genomic datasets and carefully observing the results from single gene or gene families are important.

Our present analyses with the limited crustacean samples indicate that Branchiopoda is sister to Hexapoda and that Malacostraca and Maxillopoda are their outgroups. However, recent molecular studies have strongly suggested that either Remipedia or Remipedia + Cephalocarida is most closely related to Hexapoda [11,45-47]. Therefore, the inclusion of samples from Remipedia and Cephalocarida in our future analyses is highly desirable.

### Is Entognatha monophyletic or paraphyletic?

Although numerous morphological and molecular phylogenetic analyses have been conducted to date, the relationships among the basal hexapod lineages Protura, Collembola, Diplura, and Ectognatha remain unclear (see reviews by [19,48]). The three main questions concern the following: (1) the monophyly or paraphyly of Entognatha; (2) the supposed monophyly of Ellipura (Protura + Collembola); and (3) the supposed sister relationship between Protura and Diplura, together called Nonoculata.

Entognatha and Ellipura are traditionally considered to be monophyletic mainly based on morphological features, such as “enclosed mouthparts” for Entognatha and the “absence of cerci” for Ellipura [49-51]. Molecular phylogenetic studies based on rDNA sequences [7,29,30,37] and phylogenomic dataset [8] support the monophyly of Entognatha but reject the Ellipuran clade; they suggest instead a sister relationship between Protura and Diplura, called Nonoculata. One morphological feature, the lack of eyes (hence the name Nonoculata), has been cited in support of this pairing [30]. However, another recent phylogenomic analysis recovers the Ellipura with strong support [47]. In contrast to these, a Carboniferous dipluran fossil showed that only Diplura of Entognatha shares an ancestral ground plan with Ectognatha, suggesting a close relationship between Diplura and Ectognatha [32]. Comparative embryological evidence [33,34] and phylogenetic analyses based on morphological [28,35] and some molecular data (EF-1α, EF-2, and RNA polymerase II sequences) [36] also suggest that a relationship exists between Diplura and Ectognatha. Our present results reveal that Protura is the most basal lineage within Hexapoda and that Diplura or Diplura + Collembola is close to Ectognatha. In addition, the phylogenetic analyses performed without collembolans clustered Diplura and Ectognatha as a common clade with robust support (bootstrap: 94; posterior probability: 1.0) (Figure 3B). Therefore, our present results do not support either the monophyly of Entognatha or a sister relationship between Protura and Diplura (Nonoculata).

Although our results disagree with those of molecular phylogenetic analyses based on rRNA gene sequences [29,30] and EST data [8], they basically support the hypotheses inferred from comparative embryological evidence [34], dipluran fossil data [32], and morphological analyses [28,35], which indicate the paraphyly of Entognatha and a close relationship between Diplura and Ectognatha. Given that both proturans and diplurans have GC-rich rDNA sequences [52] and always show long branches in phylogenetic trees, the clustering of proturans and diplurans might be due to LBA artifacts [30,52]. On the other hand, in phylogenetic analyses based on nuclear protein-coding genes, Protura or both Protura and Diplura have been omitted [9,11,31], with the exception of one study [8], even though including these two orders is indispensable for inferring basal hexapod relationships. Meusemann et al. [8] included one proturan and one dipluran species in analyses based on EST data to resolve the arthropod phylogeny and those analyses yielded strong support for the Nonoculata. In contrast, our present analyses, which were performed with a more extensive sampling of hexapod species, strongly reject a Nonoculata clade (see Figure 3). The discrepancy between our results and those of Meusemann et al. [8] may be explained if the monophyly of Nonoculata is supported by most but not by all genes. Our results uncovered the important finding that some genes, such as DPD1, RPB1, and RPB2 clearly support a paraphyletic origin for Entognatha. These ambiguities imply that obtaining additional evidence from different molecular markers is still necessary to accurately infer deep hexapod relationships.

## Conclusion

Phylogenetic relationships among basal hexapod lineages, the knowledge of which is indispensable to understanding hexapod origins and evolution, remain ambiguous despite numerous studies. Our results, based on multiple nuclear DNA-coded protein sequences from 64 arthropod taxa, including all hexapod orders, six crustacean species (representing three classes), one myriapod, and two chelicerates with both ML and Bayesian inference analyses, support the monophyletic origin of hexapods. In addition, they reject the monophyly of Entognatha, Ellipula, and Nonoculata and suggest that Protura is a sister lineage to other hexapods and that either Diplura or Diplura + Collembola is closely related to Ectognatha. Although our results differ from those of several recent molecular studies, they basically corroborate the fossil, morphological, and comparative embryological evidence. These findings are expected to provide insights into the origin of hexapods and the processes involved in the adaptation of insects to life on land.

Within Ectognatha, our results strongly support the monophyly of the higher taxonomic groups (Ectognatha, Palaeoptera, Neoptera, Polyneoptera, and Holometabola) of hexapods. The interordinal relationships of Holometabola are also well resolved. However, the relationships among the higher groups of Neoptera and most interordinal relationships within Polyneoptera and Paraneoptera are still unresolved and require further study.

## Methods

### Samples

A total of 64 species, representing all hexapod orders, three Crustacea classes (Branchiopoda, Malacostraca, and Maxillopoda), one myriapod, and two chelicerates, were used in this study. For 14 of these species, three nuclear genes (DPD1, RPB1, and RPB2) were sequenced in this study (Additional file 1). The sequence data for other species had been determined in our previous study [39], except for five species, for which the sequence data were retrieved from published sequence databases (Additional file 1).

### RNA extraction, reverse transcription, PCR, and sequencing

Living specimens were used for RNA extraction using Isogen reagent (Nippon Gene). Depending on the body size of the sample specimen, total RNA was extracted from either a part of the specimen, such as the legs and antennae, or the whole body. The total RNA was reverse-transcribed to cDNA using the SMART RACE cDNA Amplification Kit (Clontech) and SuperScript III Reverse Transcriptase (Invitrogen). The cDNAs were then used as templates for PCR amplification with LA-Taq (Takara Bio Inc.) using degenerate sense and antisense primers for the three target genes, DPD1, RPB1, and RPB2, as previously described [39] (Additional files 2, 3, 4 and 12). First, multiple sets of sense and antisense degenerate primers for each gene were used to amplify fragments of the gene using the cDNA as a template. The PCR products of the expected size were purified and sequenced directly using an ABI 3130xl Genetic Analyzer (Applied Biosystems). Second, to amplify the 3’ and 5’ ends of the cDNA, sense and antisense gene specific primers (GSP) were designed based on the sequences obtained from the PCR products described above and then used for the 3’ and 5’ Rapid Amplification of cDNA Ends (RACE) using a SMART RACE cDNA Amplification Kit (Clontech). The PCR products were sequenced using the same method described above. Finally, the full-length coding sequences (CDS) were amplified by GSP primer sets that were designed according to the sequences of the 3’ and 5’ ends of each cDNA, and sequenced as above. The complete or nearly complete sequences of the cDNAs of the three genes were obtained from all 64 species used in this study (Additional files 2, 3, 4). These experimental procedures were performed according to Ishiwata et al. [39]. The nucleotide sequences reported in this study have been deposited in the DDBJ/EMBL/GenBank nucleotide sequence databases under the accession numbers (AB596891-AB596934, AB597582-AB597625, AB598692-AB598735, AB811978-AB812019) shown in Additional file 1.

### Sequence alignment and phylogenetic tree inference

The aa sequences of the three nuclear genes were aligned by MAFFT L-INS-i [40] and then manually inspected. Unambiguously aligned aa sites (DPD1, 873 aa; RPB1, 1401 aa; and RPB2, 1126 aa; total, 3400 aa) were selected using Gblocks ver. 0.91b with default parameters (minimum number of sequences for a conserved position: 33; minimum number of sequences for a flanking position: 54; maximum number of contiguous nonconserved positions: 8; minimum length of a block: 10; allowed gap positions: none; use similarity matrices: yes) [41,42]. The phylogenetic trees were inferred using the ML method and Bayesian analysis. For the ML analyses on RAxML 7.2.8 [53], model testing was conducted with ProtTest 3 under the AIC, BIC, and AICc criteria [54], and all criteria selected the LG model as the best-fit model. Tree searching and bootstrapping were conducted simultaneously on the RAxML program under PROTCATLGF and 1000 bootstrap replicates. Bayesian inference was performed by MCMC analysis using MrBayes v3.1.2 [55] with the WAG model. Each analysis was run for 2,000,000 generations, and the tree was sampled every 1000 generations (burn-in = 500,000 generations). To test the convergence of chains, the log file of the MrBayes analyses was examined by calculating the effective sample sizes of all parameters using Tracer v1.5 [56]. Bayesian posterior probabilities were obtained from the majority-rule consensus tree sampled after the initial burn-in period.

### Availability of supporting data

The sequence alignments for tree construction have been deposited in the TreeBASE with accession URL (http://purl.org/phylo/treebase/phylows/study/TB2:S14526).

## Abbreviations

DPD1: DNA polymerase delta catalytic subunit; RPB1: RNA polymerase II largest subunit; RPB2: RNA polymerase II second largest subunit; ML: Maximum likelihood; CDS: Coding sequences; PCR: Polymerase chain reaction.

## Competing interests

The authors declare that they have no competing interests.

## Authors’ contributions

TM and ZHS conceived the study. GS and ZHS designed the experimental strategy. GS and KI determined the sequences and performed all analyses. RM contributed some specimens and identified several species. GS and ZHS wrote the paper with input from the other authors. All authors read and approved the final manuscript.

## Supplementary Material

Additional file 1 A list of the taxa used for the phylogenetic analyses in this study. Click here for file

Additional file 2 **The coding sequence (CDS) region of the catalytic subunit of DNA polymerase delta (DPD1) sequenced in this study.** The lengths of the gene CDS are shown in accordance with those of *Drosophila melanogaster*. The locations of the primers used for amplifying and sequencing DPD1 are indicated on the gene CDS of *D. melanogaster*. The number above or below the primer name indicates the primer’s position in the nucleotide sequence following the initiation codon. The primer names correspond to those shown in Additional file 12. Click here for file

Additional file 3 **The CDS region of the largest subunit of RNA polymerase II (RPB1) sequenced in this study.** For details, refer to Additional file 2. Click here for file

Additional file 4 **The CDS region of the second largest subunit of RNA polymerase II (RPB2) sequenced in this study.** For details, refer to Additional file 2. Click here for file

Additional file 5 **The ML trees inferred from the individual genes of 64 samples (complete sample set).** Bootstrap values from the RAxML analysis (LG model) are shown at the nodes. A, DPD1 tree; B, RPB1 tree; C, RPB2 tree. Click here for file

Additional file 6 **The ML trees inferred from the individual genes of 61 samples (55 hexapods and 6 crustaceans).** Bootstrap values from the RAxML analysis (LG model) are shown at the nodes. A, DPD1 tree; B, RPB1 tree; C, RPB2 tree. Click here for file

Additional file 7 **The ML tree inferred from DPD1, RPB1, and RPB2.** The eight taxa that showed long-branches in Figure 2 were excluded in this analysis. The bootstrap values from the RAxML analysis and posterior probabilities from the MrBayes are shown at nodes. Click here for file

Additional file 8 RAxML tree with 61 samples (55 hexapods and 6 crustaceans). Click here for file

Additional file 9 Bayesian inference (MrBayes) with 61 samples (55 hexapods and 6 crustaceans). Click here for file

Additional file 10 RAxML tree with 58 samples, consisting of 52 hexapods (excluding collembolans) and 6 crustaceans. Click here for file

Additional file 11 Bayesian inference (MrBayes) with 58 samples, consisting of 52 hexapods (excluding collembolans) and 6 crustaceans. Click here for file

Additional file 12 The list of primers used for PCR and sequencing in this study. Click here for file
