# Supplementary material for: Molecular phylogenetic analyses support the monophyly of Hexapoda and suggest the paraphyly of Entognatha
Source: BMC Evol Biol. 2013 Oct 31;13:236. doi: 10.1186/1471-2148-13-236 (PMC4228403; doi:10.1186/1471-2148-13-236)
Supplement: Additional file 2 — The coding sequence (CDS) region of the catalytic subunit of DNA polymerase delta (DPD1) sequenced in this study. The lengths of the gene CDS are shown in accordance with those of Drosophila melanogaster. The locations of the primers used for amplifying and sequencing DPD1 are indicated on the gene CDS of D. melanogaster. The number above or below the primer name indicates the primer’s position in the nucleotide sequence following the initiation codon. The primer names correspond to those shown in Additional file 12. [file 1471-2148-13-236-S2.pdf]

CDS of DNA polymerase δ catalytic subunit (DPD1)

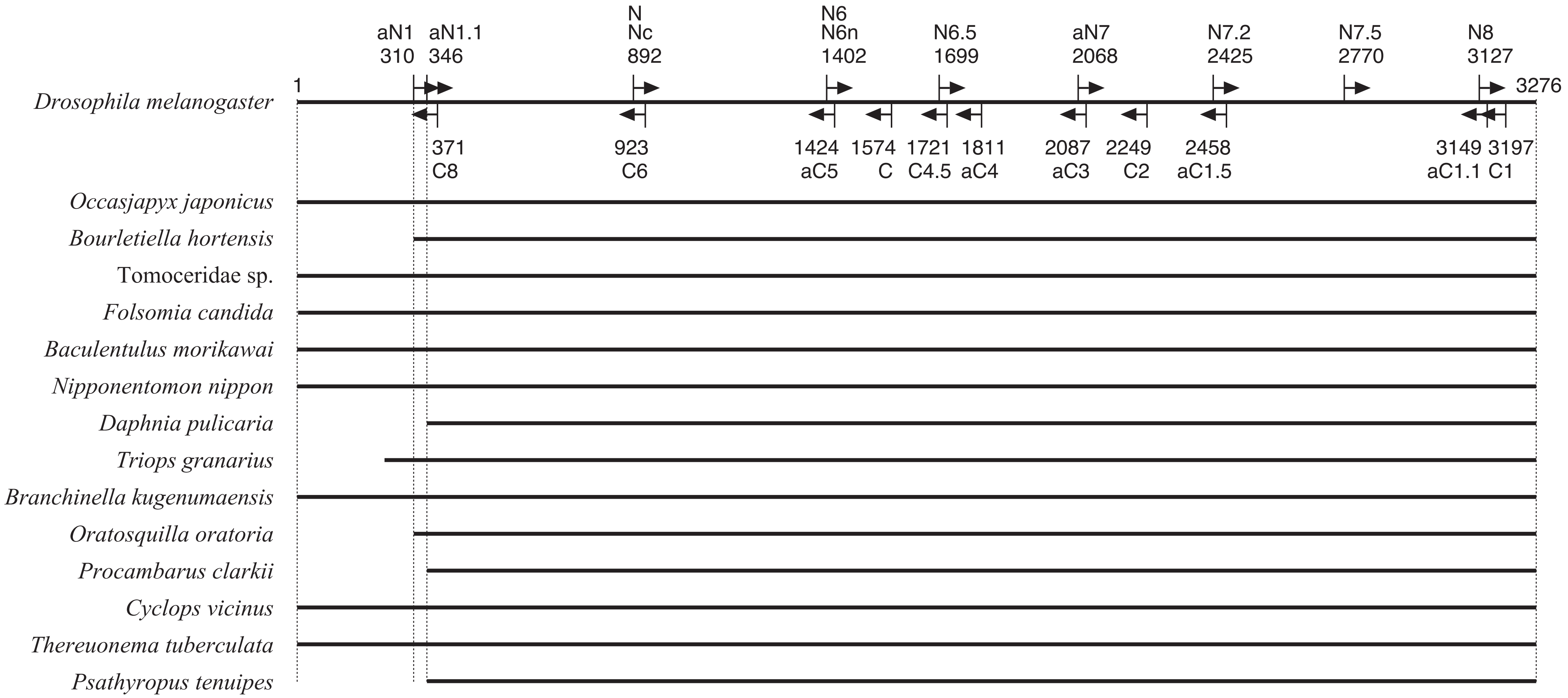

Additional file 2. The coding sequence (CDS) region of the catalytic subunit of DNA polymerase delta (DPD1) sequenced in this study. The lengths of the gene CDS are shown in accordance with those of *Drosophila melanogaster*. The locations of primers used for amplifying and sequencing are indicated on the gene CDS of *D. melanogaster*. The number above or below the primer name indicates the primer's position in the nucleotide sequence following the initiation codon. The primer names correspond to those shown in Additional file ~~17~~.
