# Supplementary material for: Molecular phylogenetic analyses support the monophyly of Hexapoda and suggest the paraphyly of Entognatha
Source: BMC Evol Biol. 2013 Oct 31;13:236. doi: 10.1186/1471-2148-13-236 (PMC4228403; doi:10.1186/1471-2148-13-236)
Supplement: Additional file 3 — The CDS region of the largest subunit of RNA polymerase II (RPB1) sequenced in this study. For details, refer to Additional file 2. [file 1471-2148-13-236-S3.pdf]

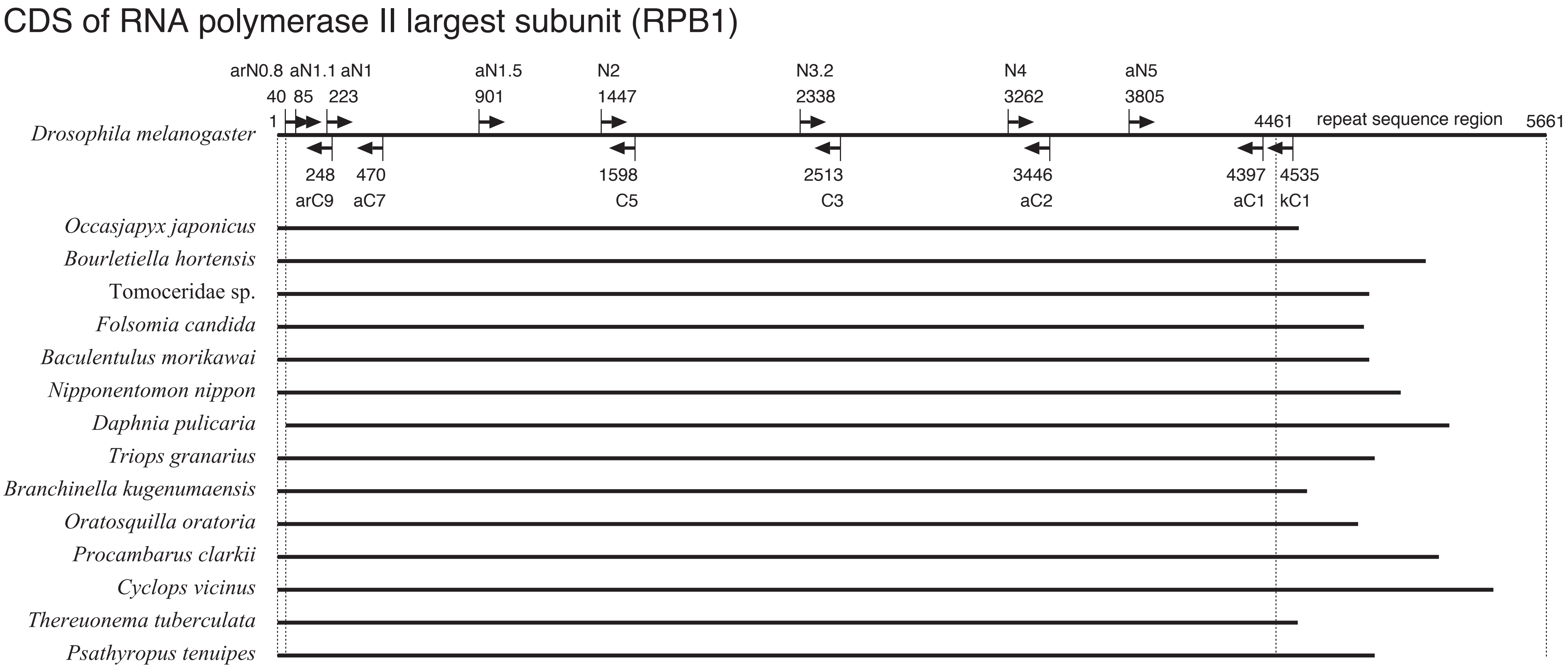

Additional file 3. The CDS region of the largest subunit of RNA polymerase II (RPB1) sequenced in this study. For details, refer to Additional file 2.
