# Supplementary material for: Molecular phylogenetic analyses support the monophyly of Hexapoda and suggest the paraphyly of Entognatha
Source: BMC Evol Biol. 2013 Oct 31;13:236. doi: 10.1186/1471-2148-13-236 (PMC4228403; doi:10.1186/1471-2148-13-236)
Supplement: Additional file 5 — The ML trees inferred from the individual genes of 64 samples (complete sample set). Bootstrap values from the RAxML analysis (LG model) are shown at the nodes. A, DPD1 tree; B, RPB1 tree; C, RPB2 tree. [file 1471-2148-13-236-S5.pdf]

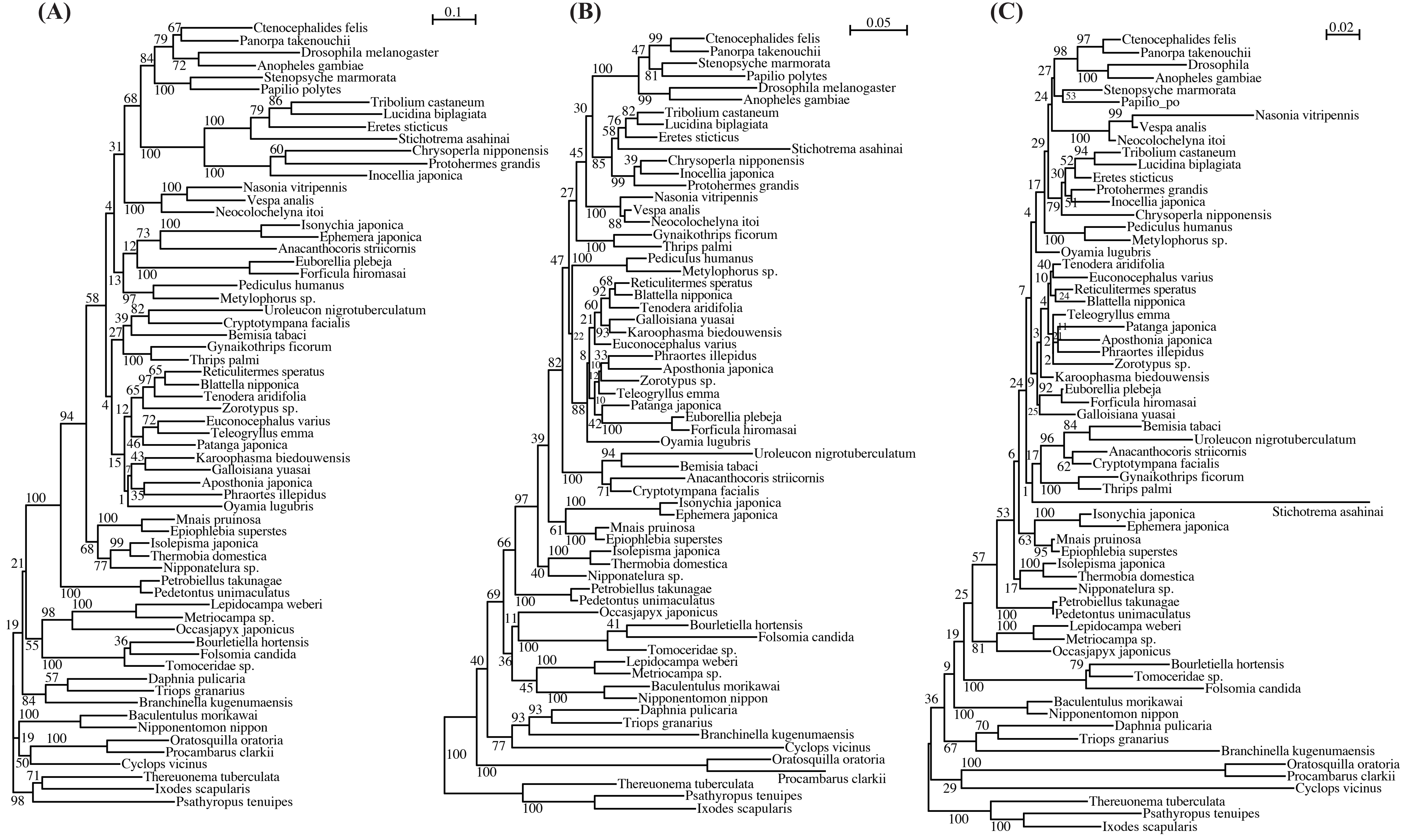

Additional file 5. The ML trees inferred from the individual genes of 64 samples (complete sample set). Bootstrap values from the RAxML analysis (LG model) are shown at nodes. A, DPD1 tree; B, RPB1 tree; C, RPB2 tree.
