# Supplementary material for: Molecular phylogenetic analyses support the monophyly of Hexapoda and suggest the paraphyly of Entognatha
Source: BMC Evol Biol. 2013 Oct 31;13:236. doi: 10.1186/1471-2148-13-236 (PMC4228403; doi:10.1186/1471-2148-13-236)
Supplement: Additional file 6 — The ML trees inferred from the individual genes of 61 samples (55 hexapods and 6 crustaceans). Bootstrap values from the RAxML analysis (LG model) are shown at the nodes. A, DPD1 tree; B, RPB1 tree; C, RPB2 tree. [file 1471-2148-13-236-S6.pdf]

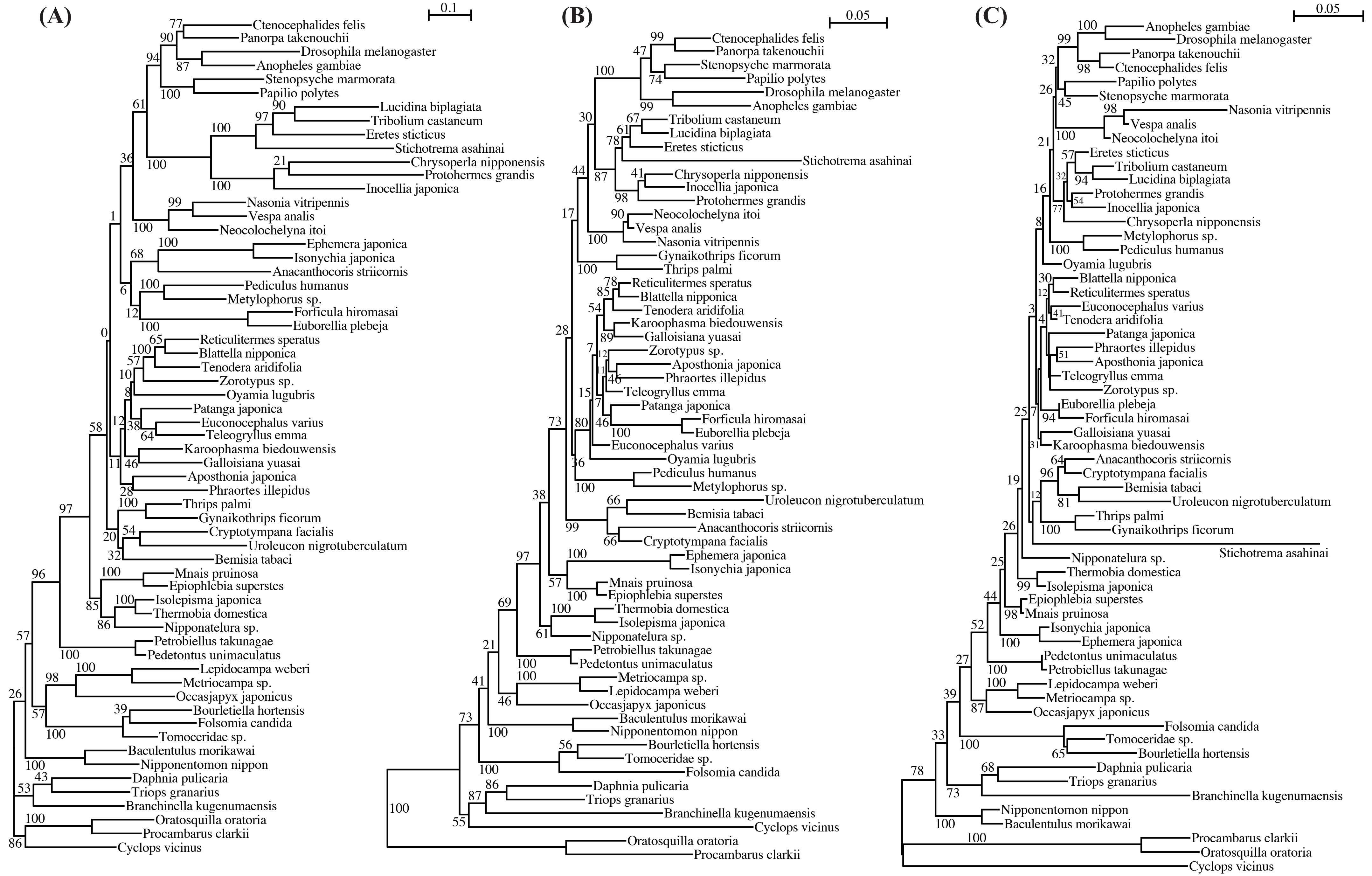

Additional file 6. The ML trees inferred from the individual genes of 61 samples (55 hexapods and 6 crustaceans). Bootstrap values from the RAxML analysis (LG model) are shown at nodes. A, DPD1 tree; B, RPB1 tree; C, RPB2 tree.

Additional file 6. Sasaki *et al*. 2013
