# Supplementary material for: Molecular phylogenetic analyses support the monophyly of Hexapoda and suggest the paraphyly of Entognatha
Source: BMC Evol Biol. 2013 Oct 31;13:236. doi: 10.1186/1471-2148-13-236 (PMC4228403; doi:10.1186/1471-2148-13-236)
Supplement: Additional file 7 — The ML tree inferred from DPD1, RPB1, and RPB2. The eight taxa that showed long-branches in Figure 2 were excluded in this analysis. The bootstrap values from the RAxML analysis and posterior probabilities from the MrBayes are shown at nodes. [file 1471-2148-13-236-S7.pdf]

**Additional file 7.** The ML tree inferred from DPD1, RPB1, and RPB2. Eight taxa that show long branches in figure 2 were excluded in this analysis. Bootstrap values from the RAxML analysis (LG model), and posterior probabilities from the MCMC analysis (WAG model) of MrBayes are shown at nodes.

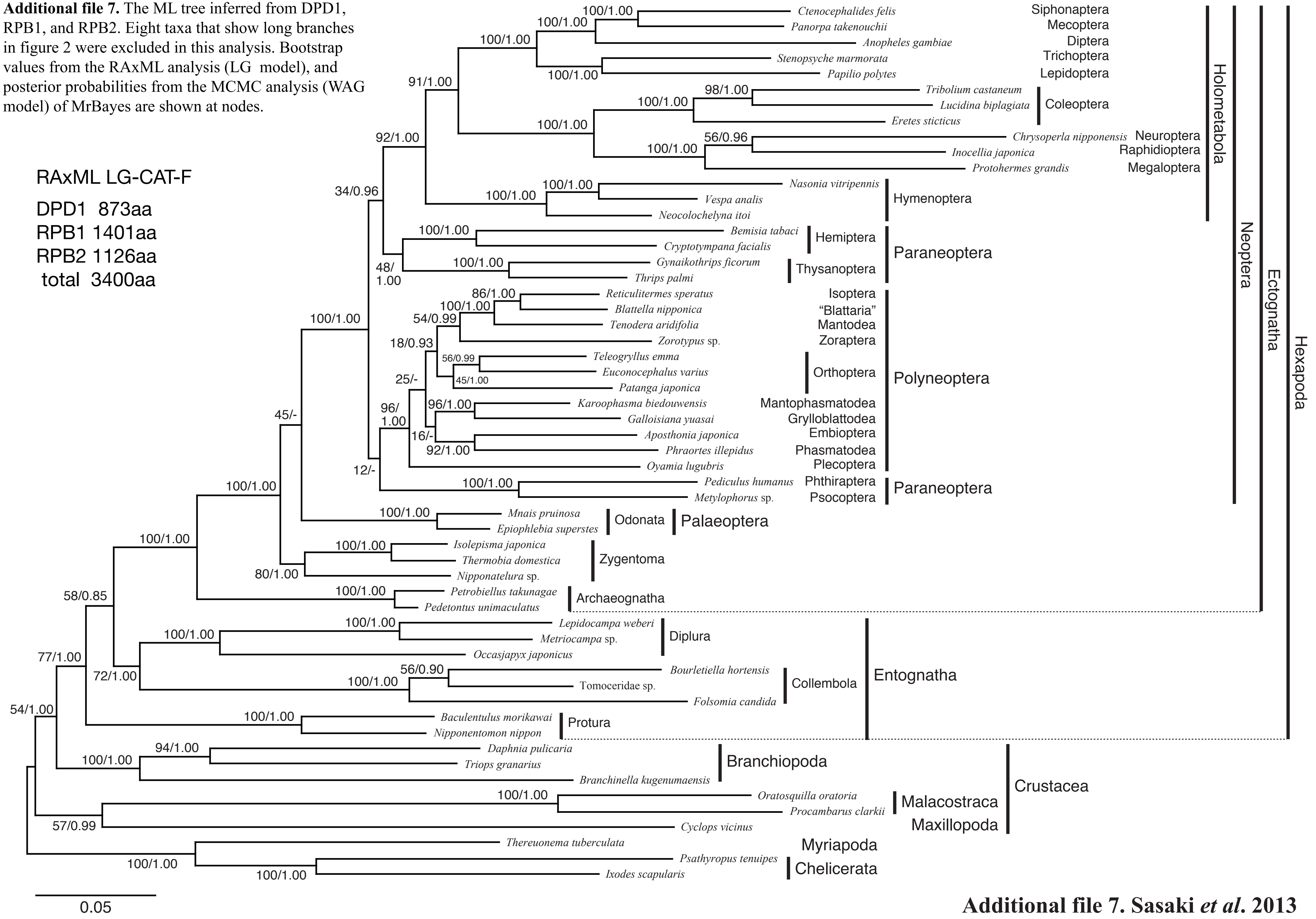


**Additional file 7. Sasaki *et al*. 2013**
