# Supplementary figures and images for: Molecular phylogenetic analyses support the monophyly of Hexapoda and suggest the paraphyly of Entognatha
Source: BMC Evol Biol. 2013 Oct 31;13:236. doi: 10.1186/1471-2148-13-236 (PMC4228403; doi:10.1186/1471-2148-13-236)

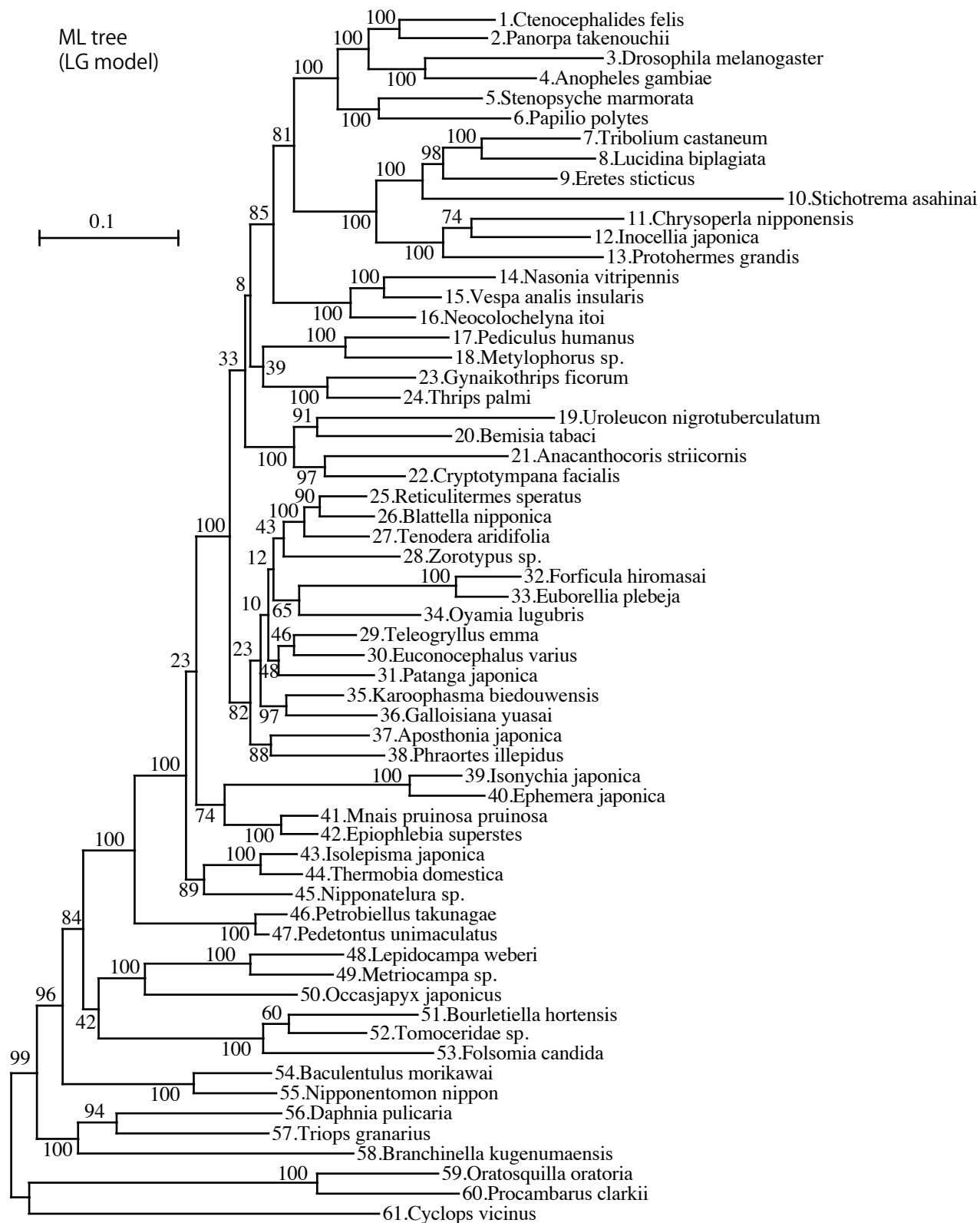

**Additional file 8.** ML tree based on LG model with 61 samples including 55 hexapods and 6 crustaceans.

Supplement: Additional file 8 — RAxML tree with 61 samples (55 hexapods and 6 crustaceans). [file 1471-2148-13-236-S8.pdf]

|       |      |
|-------|------|
| DPD1  | 872  |
| RPB1  | 1416 |
| RPB2  | 1128 |
| Total | 3416 |

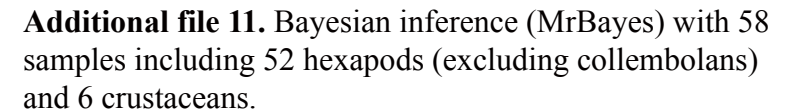

**Additional file 11. Sasaki *et al.* 2013**

Supplement: Additional file 11 — Bayesian inference (MrBayes) with 58 samples, consisting of 52 hexapods (excluding collembolans) and 6 crustaceans. [file 1471-2148-13-236-S11.pdf]
