# Supplementary material for: Molecular phylogenetic analyses support the monophyly of Hexapoda and suggest the paraphyly of Entognatha
Source: BMC Evol Biol. 2013 Oct 31;13:236. doi: 10.1186/1471-2148-13-236 (PMC4228403; doi:10.1186/1471-2148-13-236)
Supplement: Additional file 9 — Bayesian inference (MrBayes) with 61 samples (55 hexapods and 6 crustaceans). [file 1471-2148-13-236-S9.pdf]

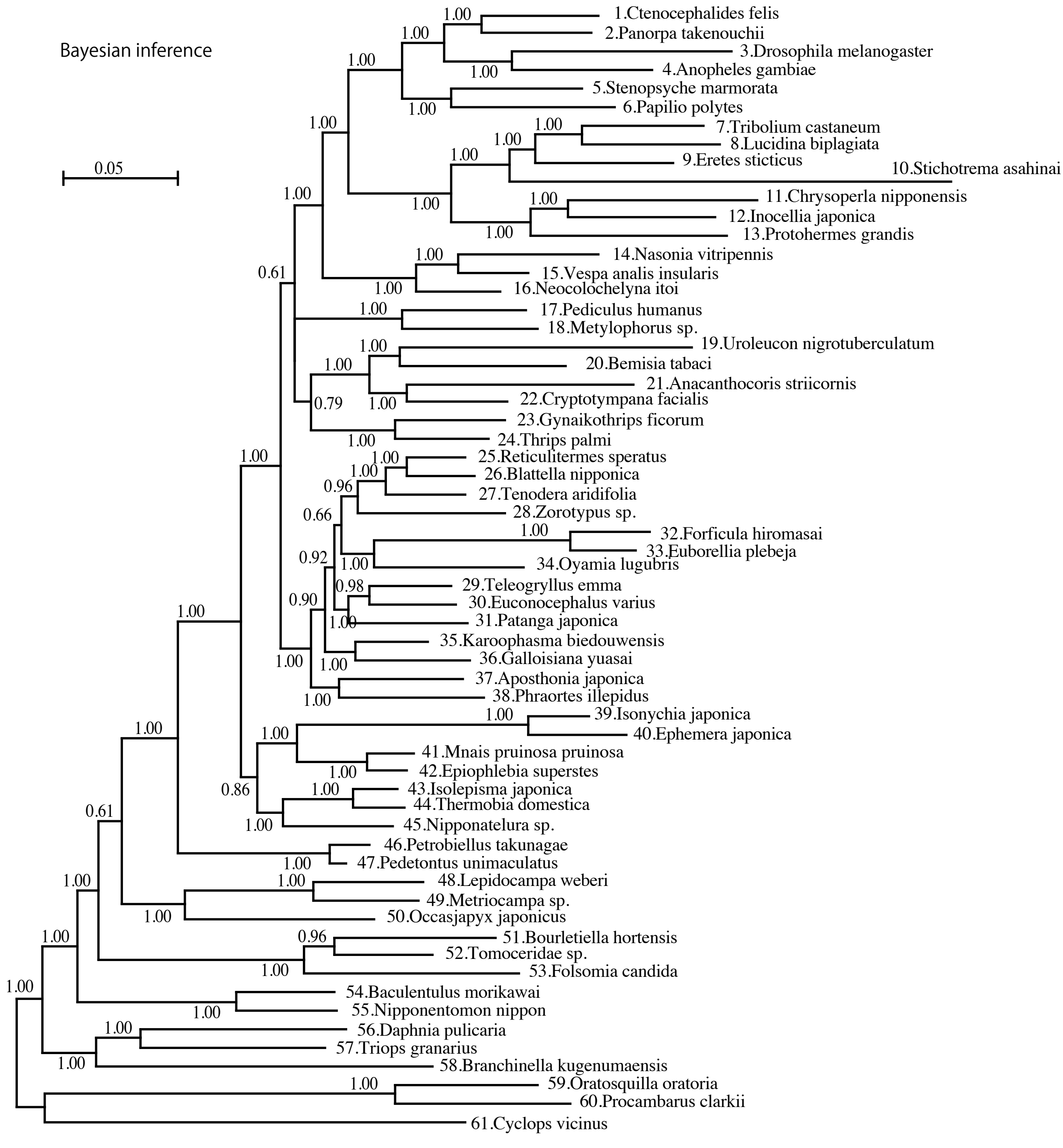

Additional file 9. Bayesian inference (MrBayes) with 61 samples including 55 hexapods and 6 crustaceans.

Additional file 9. Sasaki *et al.* 2013
