# Supplementary material for: Molecular phylogenetic analyses support the monophyly of Hexapoda and suggest the paraphyly of Entognatha
Source: BMC Evol Biol. 2013 Oct 31;13:236. doi: 10.1186/1471-2148-13-236 (PMC4228403; doi:10.1186/1471-2148-13-236)
Supplement: Additional file 10 — RAxML tree with 58 samples, consisting of 52 hexapods (excluding collembolans) and 6 crustaceans. [file 1471-2148-13-236-S10.pdf]

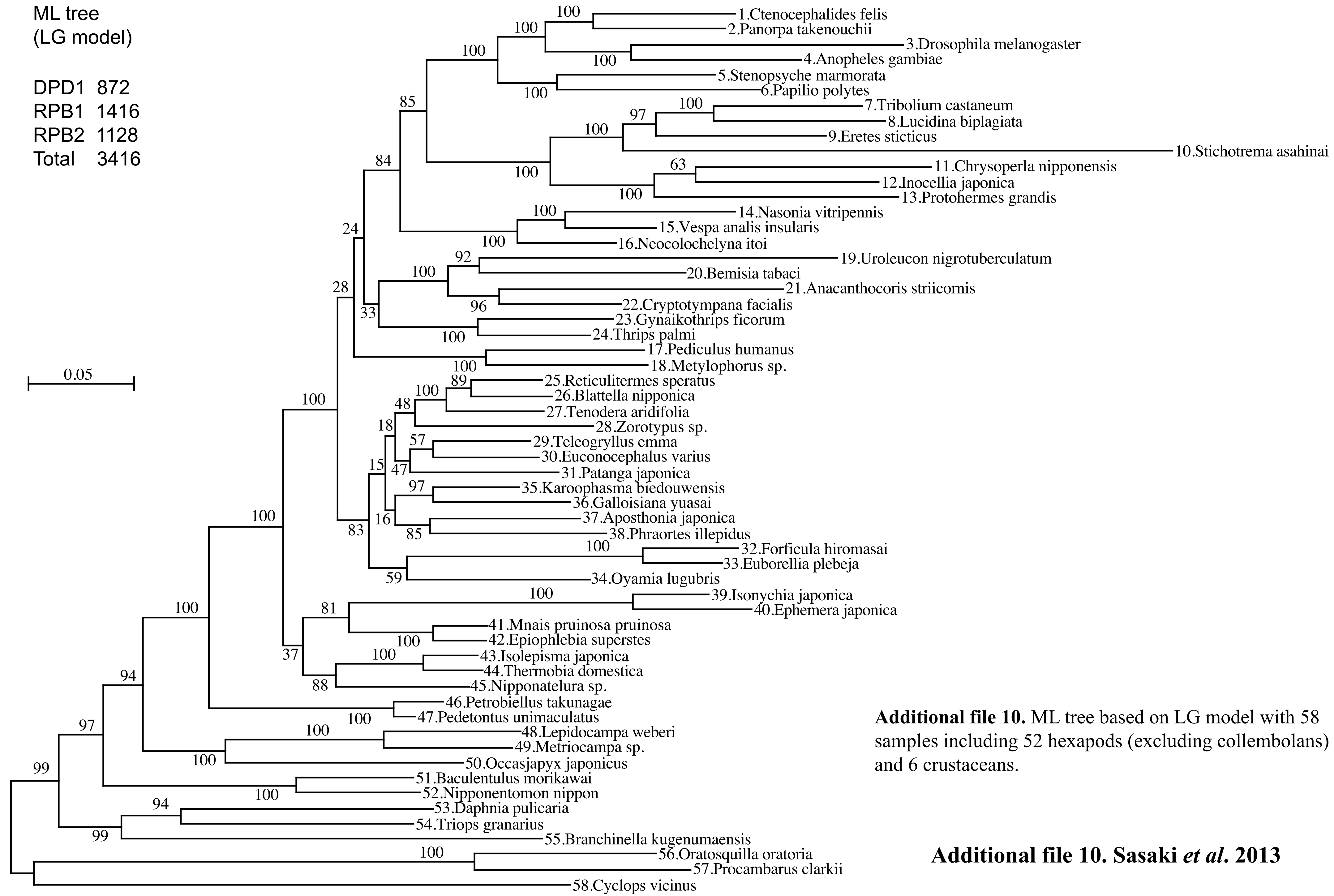

Additional file 10. ML tree based on LG model with 58 samples including 52 hexapods (excluding collembolans) and 6 crustaceans.

Additional file 10. Sasaki *et al*. 2013
